# Evaluating the Radiation Sensitivity Index and 12-Chemokine Gene Expression Signature for Clinical Use in a CLIA Laboratory

**DOI:** 10.1158/2767-9764.CRC-24-0534

**Published:** 2025-03-03

**Authors:** Anders E. Berglund, John Puskas, Sean J. Yoder, Andrew T. Smith, Douglas C. Marchion, Dahui Qin, James J. Mulé, Javier F. Torres-Roca, Steven A. Eschrich

**Affiliations:** 1 Department of Biostatistics and Bioinformatics, Moffitt Cancer Center & Research Institute, Tampa, Florida.; 2 Department of Quantitative Health Sciences, Mayo Clinic Florida, Jacksonville, Florida.; 3 Advanced Diagnostic Laboratory, Moffitt Cancer Center & Research Institute, Tampa, Florida.; 4 Molecular Genomics Core, Moffitt Cancer Center & Research Institute, Tampa, Florida.; 5 Tissue Core, Moffitt Cancer Center & Research Institute, Tampa, Florida.; 6 Department of Immunology, Moffitt Cancer Center & Research Institute, Tampa, Florida.; 7 Department of Radiation Oncology, Moffitt Cancer Center & Research Institute, Tampa, Florida.

## Abstract

The radiation sensitivity index (RSI) and 12-chemokine gene expression signature (12CK GES) are two gene expression signatures (GES) that were previously developed to predict tumor radiation sensitivity or identify the presence of tertiary lymphoid structures in tumors, respectively. To advance the use of these GESs into clinical trial evaluation, their assays must be assessed within the context of the Clinical Laboratory Improvement Amendments (CLIA) process. Using HG-U133Plus2.0 arrays, we first established CLIA laboratory proficiency. Then the accuracy (limit of detection and macrodissection impact), precision (variability by time and operator), sample type (surgery vs. biopsy), and concordance with a reference laboratory were evaluated. RSI and 12CK GES were reproducible (RSI: 0.01 mean difference, 12CK GES: 0.17 mean difference) and precise with respect to time and operator. Taken together, the reproducibility analysis of the scores indicated a median RSI difference of 0.06 (6.47% of range) across samples and a median 12CK GES difference of 0.92 (12.29% of range). Experiments indicated that the lower limit of input RNA is 5 ng. Reproducibility with a second CLIA laboratory demonstrated reliability with the median RSI score difference of 0.065 (6% of full range) and 12CK GES difference of 0.93 (12% of observed range). Overall, under CLIA, RSI and 12CK GES were demonstrated by the Moffitt Cancer Center Advanced Diagnostic Laboratory to be reproducible GESs for clinical usage.

**Significance:**

The RSI and 12CK GES are two GESs that predict tumor radiation sensitivity or the presence of tertiary lymphoid structures in tumors, respectively. These GESs were assessed within the CLIA process for future clinical use. We established proficiency, reproducibility, and reliability characteristics for both signatures in a controlled setting, indicating these GESs are suitable for validation within future clinical trials.

## Introduction

Molecular gene expression signatures (GES) have been developed for multiple purposes within cancer research and for potential use in clinical oncology indications across different tumor types, such as breast (1) and prostate cancers (2). Many studies use clinical specimens for identifying molecular differences with respect to patient outcome or treatment response. With the advent of several clinically validated gene expression array platforms, new GESs can be derived from data generated from these platforms. Far fewer publications exist for evaluating specific GES for clinical decision-making and/or clinical trial designs in a Clinical Laboratory Improvement Amendments (CLIA) laboratory setting. The moving of a molecular GES from research-grade data to a reproducible, clinical GES involves experimental evaluation of multiple analytic variables, including sample type and RNA quality (3). Technical variability is common in molecular GES research studies (4). However, this variability can be explicitly evaluated in the context of a clinical laboratory that generates the necessary operating characteristics for molecular gene expression tests to be used under clinical conditions. Without adequate assessment of technical variability, clinical validation as a necessary step within the context of a clinical trial would not be expected to be successful.

The radiation sensitivity index (RSI) is a molecular signature to predict tumor sensitivity to radiotherapy. RSI was developed from a subset of the NCI-60 cell lines that were profiled using Affymetrix Hu6800 gene expression arrays (5). The survival fraction at 2 Gy was used with baseline gene expression levels to model an association between gene expression and radiation response. Ten hub genes from among the significant gene expression results were identified using a network biology approach (6). These 10 genes were combined into a simple rank-based linear model for predicting RSI. This model was demonstrated to predict clinical outcome in patient cohorts (6). Subsequently, RSI has been used to assess clinical outcome in several cancer types, and its radiation-specific nature is indicated in cohorts in which RSI is prognostic only in radiotherapy-treated patients (7–16). Importantly, the RSI model originally developed in 2009 has not been modified from the original formula. RSI was used together with dose information to calculate the genomically adjusted radiation dose (17) to estimate the effect of radiation on a tumor by using the linear quadratic model. Genomically adjusted radiation dose has been demonstrated to predict for radiation response more accurately than RSI alone in several diseases, including breast and lung cancers (18–20). Table 1 highlights the major studies that demonstrated the clinical utility of RSI in retrospective clinical cohorts.

**Table 1:** Publications on the use of RSI/12CK GES in retrospective cohorts

Disease	Reference
RSI	
Breast cancer	Eschrich and colleagues (7); Torres-Roca and colleagues (10); Sjöström and colleagues (12); Kang and colleagues (15)
Prostate cancer	Thiruthaneeswaran and colleagues (27)
Colon cancer	Ahmed and colleagues (28)
Sarcoma	Yang and colleagues (29)
Penile cancer	Johnstone and colleagues (30)
Pancreatic cancer	Strom and colleagues (31)
Glioblastoma	Ahmed and colleagues (9)
Lung cancer	Scott and colleagues (20)
Endometrial cancer	Mohammadim and colleagues (14)
Bladder cancer	Khan and colleagues (32)
Melanoma	Strom and colleagues (11)
Pan-cancer	Scott and colleagues (17, 19); Strom and colleagues (31); Ahmed and colleagues (33, 34)
12CK GES	
Colorectal carcinoma	Coppola and colleagues (21)
Pan-cancer/melanoma	Messina and colleagues (22)
Breast cancer	Prabhakaran and colleagues (35)
Bladder cancer	Li and colleagues (36)
The Cancer Genome Atlas	Li and colleagues (37)
Clear-cell renal cell carcinoma	Xu and colleagues (38)

The 12-chemokine gene expression signature (12CK GES; *CCL2*, *CCL3*, *CCL4*, *CCL5*, CCL8, *CCL18*, *CCL19*, *CCL21*, *CXCL9*, *CXCL10*, *CXCL11*, and *CXCL13*) was first developed in a large cohort of colorectal cancer samples (21) and showed a strong correlation between the 12CK GES and the presence of ectopic lymph node–like/tertiary lymphoid structures (TLS; ref. 22). The 12CK GES was derived from a set of related chemokines that demonstrated its predictive ability for immunotherapy response, better patient survival, and the presence of TLS, which has been validated in at least six cohorts (Table 1).

Given the literature support for the value of these two GESs, there is translational oncology interest in evaluating them in prospective clinical trials to determine their clinical utility. To do so, the operating characteristics of the RSI/12CK GES, above and beyond the platform characteristics, must be established. Therefore, we undertook a comprehensive series of experiments to establish these operating characteristics and validation in a CLIA-certified laboratory (i.e., the Moffitt Advanced Diagnostics Laboratory).

## Methods

### Sex as a biological variable

This study was a technical study of the reproducibility of a molecular diagnostic in a clinical laboratory. Therefore, we did not consider sex as a biological variable.

### Ethics approval and consent to participate

Informed consent was obtained from patients to the Total Cancer Care (TCC) protocol, the Moffitt Cancer Center’s (MCC) institutional biorepository. This study was performed in accordance with the U.S. Common Rule (45 CFR 46). The Total Cancer Care protocol was approved by the Advarra Institutional Review Board: MCC #14690; Advarra Institutional Review Board Pro00014441. Tissue experiments were determined to be nonhuman subject research by MCC under the cancer center’s biospecimen pilot project process.

### Tissue samples

Tissue samples were assayed under various conditions using the HG-U133Plus 2.0 GeneChip between April 02, 2019, and August 17, 2020. Samples were selected based on diagnosed disease and tissue quantity available. The Affymetrix GeneChip Scanner 3000 7G (RRID: SCR_019341) was used for MCC experiments. Unless indicated in the experiment, the evaluated samples were generated from 100 ng input RNA and 15 μg cRNA. The Thermo Fisher Scientific GeneChip 3′ IVT PLUS Reagent Kits were used for sample processing.

Colorectal cancer samples (*n* = 10) were blinded in this study; however, the anatomic site of collection consisted of colon not otherwise specified (NOS; *n* = 6), transverse colon (*n* = 1), cecum NOS (*n* = 1), sigmoid colon (*n* = 1), and splenic flexure (*n* = 1). Similarly, the head and neck cohort (*n* = 30) was blinded but consisted of larynx (*n* = 4), maxilla (*n* = 1), oral cavity (*n* = 3), soft tissue (*n* = 1), tongue (*n* = 17), and tonsils (*n* = 4). The breast cancer cohort (*n* = 4) consisted of three breast cancer samples and one prostate cancer sample.

The most relevant experiments are presented as results and detailed in Table 2. Note that the same GeneChip may be reused in different experiments (e.g., variation over time and operator to operator) as appropriate. Supplementary Table S1 includes the specific samples used for each experiment with the corresponding GES (RSI and 12CK GES) values. Tissue/RNA assessment values are included in Supplementary Table S2.

**Table 2:** Experimental design of CLIA validation experiments. The experiments were organized into three categories: accuracy, precision, and sample content. Each of these categories assessed different sources of variability expected to impact signature scores

Study	*N*	Samples	Category	Source^a^	Goal
Proficiency	8	Four samples × two laboratories	Accuracy	CRC	Determine proficiency of the CLIA laboratory by comparing samples from research laboratory vs. CLIA laboratory
Repeatability	16	Four samples × four replicates	Precision	CRC	Determine repeatability of the signature using four replicates for each of four samples run on the same day from the same operator
Operator	12	Three samples run in duplicate by two operators	Precision	CRC	Determine impact of two different operators
Time	8	Four samples run in duplicate 1 week apart	Precision	CRC	Determine impact of processing replicates 1 week apart
LOD	17	Three samples were assessed at five different amounts of input RNA: 100, 25, 10, 5, and 2.5 ng^b^	Accuracy	CRC	Determine a lower threshold of input RNA for successful signature generation
Macrodissection	9		Accuracy	HNC	
Surgery vs. biopsy (BRCA)	8	Four samples × two sample types	Sample	BRCA	Assess the variability due to sample preparation
			Content		
Surgery vs. biopsy (HNC)	10	Five samples × two sample types	Sample	HNC	Assess the variability due to sample preparation
			Content		
MCC vs. external laboratory	28	30 samples attempted × two laboratories^c^	Concordance	HNC	Assess variability from multiple CLIA laboratories

a BRCA, breast cancer; CRC, colorectal cancer; HNC, head and neck cancer; LOD, limit of detection.

b S19/2.5 ng was lost when prepared for hybridization.

c Seven samples failed processing steps; nine excluded because of poor QC (scaling factor *>*1 and percent present *<*40%).

#### Proficiency study

Four samples previously profiled in a research genomics facility were profiled in the MCC CLIA laboratory.

#### Surgery versus biopsy study

We evaluated four breast cancer samples and five head and neck cancer samples. Core biopsies were obtained from the corresponding tissue block to simulate a clinical biopsy.

#### Concordance study

Thirty frozen tumor specimens (head and neck cancer surgical samples) were profiled in the MCC CLIA laboratory and an external provider (CLIA Outside Validation). RNA was extracted via the flow-through for DNA extraction on the QIAcube using the QIAGEN AllPrep DNA/RNA/miRNA Universal kit. RNA integrity was assessed using the TapeStation. The attrition of subjects was based on both their tissue and array quality, using the following filtering criteria: MAS5.0 scaling factor ≤1, percent present ≥40, and RNA integrity number (RIN) ≥6.5. See Supplementary Table S2 for details on RIN values for all samples.

#### The 12CK GES

For the 12CK GES, Brainarray36 HG-U133Plus2 Hs ENTREZG.cdf version 25.0.0 downloaded from http://mbni.org/customcdf/25.0.0/entrezg.download/HGU133Plus2_Hs_ENTREZG_25.0.0.zip on February 25, 2022, was used. The following probesets were used for each of the 12CK genes: *CCL2* (6347_at), *CCL3* (missing), *CCL4* (6351_at), *CCL5* (6352_at), *CCL8* (6355_at), *CCL18* (6362_at), *CCL19* (6363_at), *CCL21* (6366_at), *CXCL9* (4283_at), *CXCL10* (3627_at), *CXCL11* (6373_at), and *CXCL13* (10563_at). A principal component analysis (PCA) model was derived using the 74 samples from the GSE15605 dataset (23). The raw CEL files were downloaded and processed using IRON (24). The HG-U133Plus2 Hs ENTREZG 25.0.0 version of CDF was used. IRON was used with default settings, and GSM390277.CEL was used as a median sample. The 11 probesets were selected, and a PCA model was calculated. Principal component one (PC1) explains 64.3% of the variation, and the PC1/PC2 ratio is 5.1, indicating a robust PCA model (25). All loadings in the first component are positive, indicating that the PCA model behaves as expected.

The 12CK GES scores and RSI scores can be found in Supplementary Table S1 for each dataset/experiment.

#### RSI

Each experiment was normalized using robust multi-array average (RMA) independently using R/Bioconductor (R RRID: SCR_001905; Bioconductor RRID: SCR_006442). The 10 genes were rank-ordered per sample and RSI calculated as previously described in Eq. A. The following probesets were used for each of the RSI genes: *AR* (211110_s_at), *JUN* (201466_s_at), *STAT1* (AFFX-HUMISGF3A/M97935_MA_at), *PRKCB* (207957_s_at), *RELA* (201783_s_at), *ABL1* (202123_s_at), *SUMO1* (208762_at), *PAK2* (205962_at), *HDAC1* (201209_at), and *IRF1* (202531_at).

RSI = -0.0098009 × AR + 0.0128283 × JUN + 0.0254552 × STAT 1 -0.0017589 × PRKCB -0.0038171 × RELA + 0.1070213 × ABL1 -0.0002509 × SUMO1 -0.0092431 × PAK2 -0.0204469 × HDAC1 -0.0441683 × IRF 1 (A)

#### Statistical analysis

Data normalization and GES calculations were performed as described above either in Rv4.0 (RRID: SCR_001905) for RSI or MATLAB R2023a (RRID: SCR_001622) for 12CK GES.

Correlation coefficients were calculated using Pearson and Spearman correlation. The Passing–Bablok test used was from the mcr R package.

### Data availability

Supplementary tables contain the data used within this article. The MATLAB (R2023a The MathWorks Inc.; RRID: SCR_001622) code for generating all the figures is available at https://github.com/aebergl/12CK_RSI_Article.

## Results

### Profiling of paired samples in the research laboratory and CLIA laboratory shows high concordance (proficiency study)

**Figure 1:**
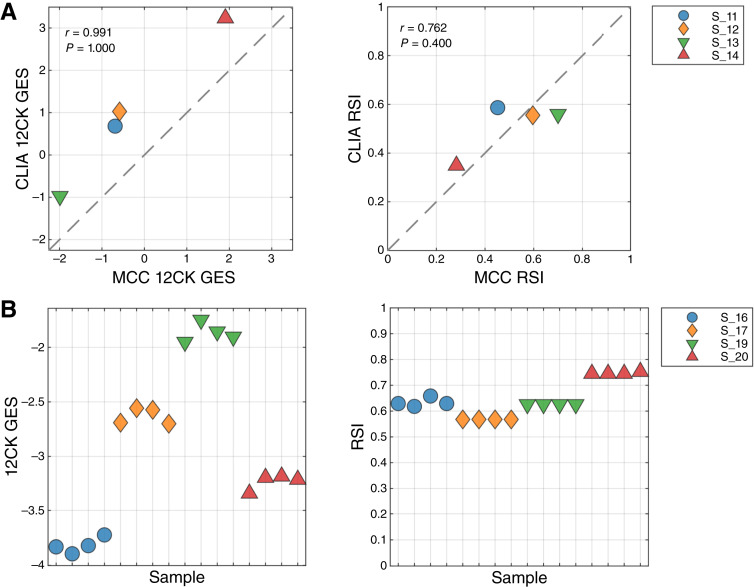
Proficiency and repeatability of the CLIA laboratory in generating 12CK GES and RSI. **A,** Proficiency of the CLIA laboratory compared with an established research-grade molecular genomics shared resource facility. Four samples were previously processed by the MCC Molecular Genomics Core (MGC) and available for CLIA laboratory processing. GESs were derived from both experimental conditions. The experiment was performed to determine that the CLIA laboratory was proficient in generating the expression data for the HG-U133Plus platform. Left, 12CK GES scores in MGC vs. CLIA laboratory (*r* = 0.991) indicating high correlation, although signature calibration was needed. Right, RSI signature scores in MGC vs. CLIA laboratory indicating compressed RSI signal from the CLIA experiments. **B,** Repeatability of GES from quadruplicate samples in the CLIA laboratory. Four samples were processed in quadruplicate and arrayed in the CLIA laboratory from the same operator. GES scores were derived from each experiment. Left, 12CK GES scores had a low variability in each of the four samples. Right, RSI was identical in two samples and had low level of variability in two samples.

Four fresh-frozen samples that were previously profiled on the same platform in a research molecular genomics shared resource facility (MCC Molecular Genomics Core) were repeated within the CLIA-certified laboratory. The gene signatures were calculated from arrays independently from the MCC Molecular Genomics Core and CLIA environments, and the correlation of the signature scores were compared (Fig. 1A). Interestingly, the 12CK GES showed a systematic shift in score range but otherwise demonstrated very high correlation (*r* = 0.991). The RSI scores showed less correlation (*r* = 0.762); however, the range of observed RSI was compressed in the CLIA experiment. These results emphasize the need to characterize the performance of each GES, as the characteristics differ even with the same samples.

### Replicated assays demonstrate repeatability of signature scores

We next profiled four samples in quadruplicate to assess the repeatability of the GES. Each sample was processed independently by the same operator, resulting in four distinct gene expression arrays per sample. As shown in Fig. 1B, the 12CK GES varied for each sample, but the overall variability was low with a mean range of scores by sample of 0.16825 (7.8% of total observed range). Likewise, the RSI score variability was generally low with a mean range of scores by sample of 0.01 (6.5% of total observed range). In the case of RSI, two samples (S19 and S17) show identical signature scores across all four replicates. The RSI is rank-based; therefore, small variations in expression do not always result in differences in GES score. Operators and time demonstrate higher RSI variability but not 12CK GES variability.

**Figure 2:**
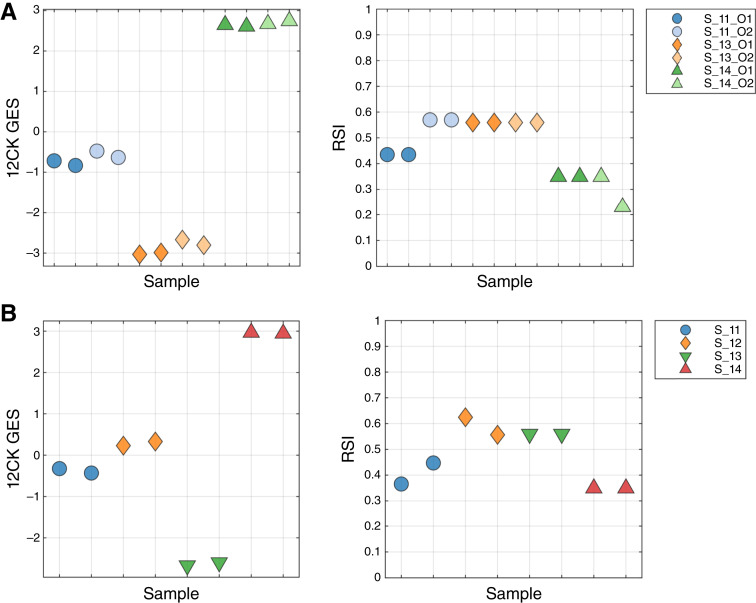
Replicability and precision analysis through measuring operator-to-operator and repeated run experiments. **A,** Operator variability: Three samples were run in duplicate by two different operators to assess both the variability in operator handling and repeatability from the same operator. The 12CK GES showed low variability across operator, whereas a larger difference in RSI score was obtained between operators. **B,** Repeated run over time: Four samples were repeated 1 week apart and assessed. The variability in the 12CK GES was low for all samples. RSI showed variability (less than 0.1) in two of the four samples.

The replicability and precision of the GES were assessed using two different experiments, evaluating the impact of operator and processing date on the GES. To test the operator characteristics, three samples were run in duplicate by two different operators (O1 and O2; Fig. 2A). The 12CK GES showed a very small operator effect, much smaller than the sample differences in GES score. In contrast, the operator had a larger impact in one sample (S11) for RSI. The impact of processing date was assessed by profiling four samples that were independently processed 1 week apart (Fig. 2B). The 12CK GES showed a low variation among replicates across time. In the case of RSI, S11 and S12 showed differences (less than 0.1 difference) in replicates, whereas the other two replicates were identical in score.

### Summary reproducibility

Using the experiments in which four replicates were produced (either from operator-to-operator variability or the repeatability study), Table 3 shows the summary mean and SD for the GES scores. Overall, reproducibility analysis of the scores indicated a median RSI difference of 0.06 (6.47% of range) across samples and a median 12CK GES difference of 0.92 (12.29% of range). The GESs are very reproducible in the CLIA laboratory using the predefined processing protocols.

**Table 3:** Summary of reproducibility of signature scores across operator-to-operator and repeatability experiments

Sample	*N*	RSI mean (SD)	12CK GES mean (SD)
11	4	0.5 (0.078)	−0.67 (0.149)
13	4	0.56 (0)	−2.87 (0.169)
14	4	0.32 (0.059)	2.67 (0.057)
16	4	0.63 (0.017)	−3.82 (0.071)
17	4	0.57 (0)	−2.63 (0.075)
19	4	0.63 (0)	−1.87 (0.087)
20	4	0.75 (0.004)	−3.23 (0.072)

In each case, four replicates of the sample were performed; listed are the mean and SD for each sample. Note that the first four samples are operator to operator.

### Amount of material (input RNA) indicates that 2.5 ng is the lower limit for GES

**Figure 3:**
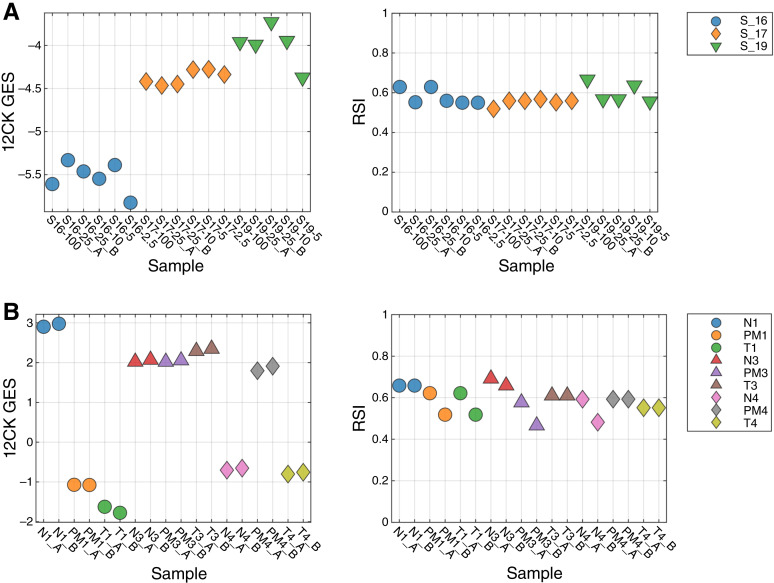
**A,** Impact of the amount of input RNA on GES scores. Three samples were profiled using different amounts of input RNA (100, 25, 10, 5, and 2.5 ng). Lower 12CK GES scores were observed at 2.5 ng, suggesting a lower limit on input RNA. RSI demonstrated more variability overall but did not seem to have a systematic difference at 2.5 ng. **B,** Impact of macrodissection on signature scores. Three samples were profiled across three different sample conditions: T, N and non-macrodissected tissue (PM). As expected, normal tissue can result in large changes to the signature score, which can be seen in PM as well. For instance, the 12CK GES score for normal tissue from sample 1 is much higher; however, there is elevated signal in the PM sample as well. By contrast, the differences in sample 3 (N, T, and PM) are small.

An important consideration for any molecular test is the amount of material required to perform reliably and robustly. To address this question, three samples were profiled using 100, 25, 10, 5, and 2.5 ng of input RNA (Fig. 3A). For the 12CK GES, the scores are very similar across RNA amounts, with a lower score observed in the 2.5-ng condition, suggesting a lower limit on RNA.

RSI demonstrated more overall variability (less than 0.1); however, it did not seem to have an input amount–related impact.

### Tissue type factors

We also assessed the impact of macrodissection or normal tissue mixtures on GES score. From three samples, we examined the tumor macrodissected tissue (T), normal macrodissected tissue (N), and non-macrodissected tissue. As shown in Fig. 3B, the N scores demonstrate large differences from T scores as is the case with the 12CK GES for S1. In this case, the non-macrodissected tissue 12CK GES score was more similar to the corresponding N score. In other cases, the RSI score (S3) showed a large difference between N and T scores. These results indicate that, as expected, GES scores derived from tumor tissue must be assayed from macrodissected (predominantly) tumor tissue to be reliably reproduced.

### Surgical specimens versus punch biopsies in breast and head and neck cancers introduces variability

**Figure 4:**
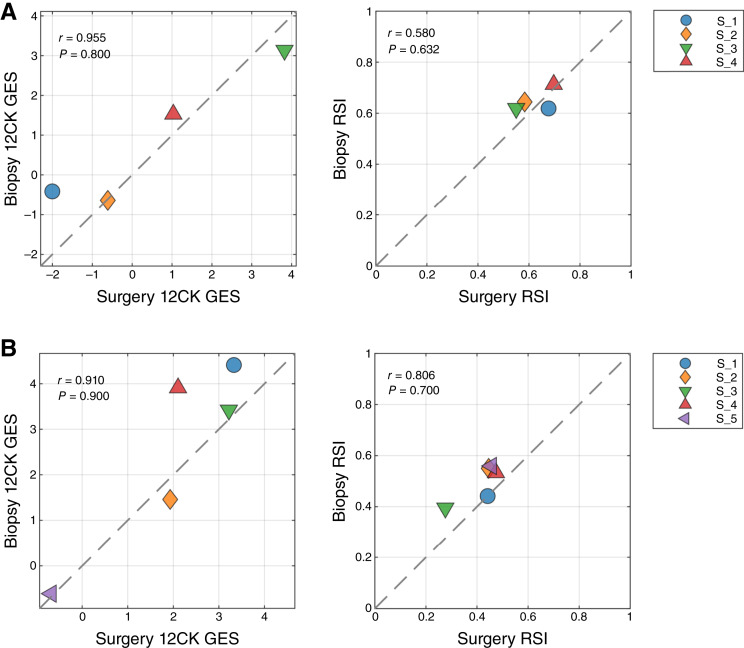
Impact of surgery vs. biopsy sample on signature scores. **A,** Breast cancer specimens from tissue resection (surgery) and punch biopsy (biopsy) were compared. **B,** Head and neck cancer specimens from tissue resection (surgery) and punch biopsy (biopsy) were compared.

Although both GESs were developed from macrodissected surgical specimens, punch biopsies are often a more readily available source of material for clinical assays. Therefore, we assessed the variability in GES scores between these preparation types in two different diseases: breast and head and neck cancers. Core biopsies were obtained from tissue blocks. Fig. 4A indicates that in breast cancer, the 12CK GES score can be attenuated by punch biopsy, whereas the RSI score showed small changes in both directions. In the case of head and neck cancer, both the 12CK GES and RSI scores showed increases in scores for biopsy samples (Fig. 4B). This may relate to the cellular content and/or proportion of immune cells present in the specimen. The variability introduced into the scores due to preparation type can be used when considering alternative methods for use. Interestingly, RSI tended to have a much narrower range of values (e.g., 0.6–0.8 for breast cancer vs. 0.2–0.5 head and neck cancer), indicating some tissue-specific sensitivity that has been noted previously.

### Concordance with an external laboratory

**Figure 5:**
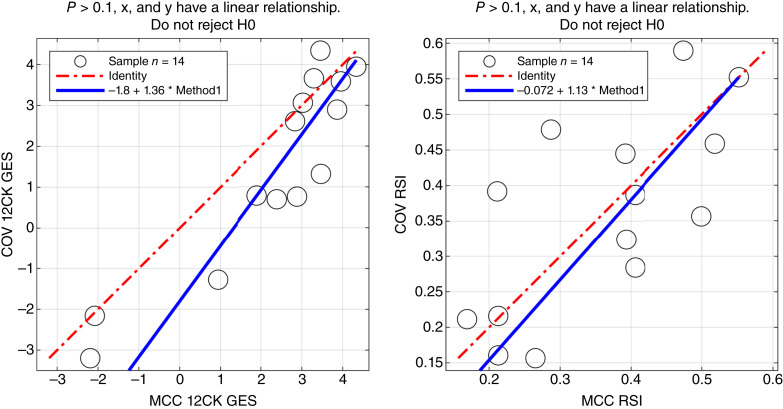
Concordance of signature scores across MCC CLIA laboratory and external vendor. Thirty samples were profiled in the MCC CLIA laboratory and external vendor. After excluding low-quality samples, the linearity of the 12CK GES (left) and RSI (right) scores for 14 samples across sites was assessed. COV, CLIA Outside Validation.

Thirty samples were processed internally at the MCC CLIA laboratory and sent to an external vendor for processing. Five samples failed processing and were not hybridized; two samples were hybridized but failed initial quality control (QC). An additional nine samples were excluded because of poor QC. After filtering, GES scores from 14 samples were compared between the MCC CLIA laboratory and the external vendor (Fig. 5). Using the Passing–Bablok test (26), both the 12CK GES and RSI signatures had linear relationships between the two sites. The median RSI score difference was 0.065 (6% of full range), and the 12CK GES difference was 0.93 (12% of observed range).

## Discussion

We describe a series of experiments that were performed to systematically assess the assay platform (the Affymetrix HG-U133Plus 2.0 GeneChip) and specific gene signatures (RSI and 12CK GES) for CLIA laboratory use in clinical trials assessing utility. Our results indicate that specific GESs have different operating characteristics, even using the same platform and hybridizations. Thus, it is important for each GES to undergo systematic evaluation for its own unique precision and robustness limits.

The studies described herein were initially performed to provide support for the CLIA validation of RSI. We note that a “valley of death” exists for GES being deployed clinically. Retrospective analyses across a variety of platforms are more typically observed in research studies; however, clinical translation requires explicit experiments to clearly assess the operating characteristics under controlled conditions. Importantly, the arrays generated in this study were subsequently reused to assess the variability in the 12CK GES without requiring additional hybridizations. As new GESs are developed, this dataset can support rapid verification of any additional GES under controlled conditions. These types of experiments systematically generated across clinical specimens as a public resource can provide initial data on the reliability of the GES for clinical translation, in particular in which patient decisions are made.

One aspect for GES is the underlying model used for prediction. RSI, for instance, was developed as a rank-based linear regression to maximize robustness across multiple platforms. This approach has been used for validation in many retrospective cohorts. Notwithstanding, the model was not designed to optimize precision. This can be seen in the evaluation for CLIA operations; the level of variability observed for RSI is higher than that observed for the 12CK GES for this reason. In contrast, the 12CK GES was developed as a PCA-based model and is likely more robust to individual genes introducing variability. In both cases, the research-grade model has been used for extensive retrospective validation in clinical cohorts. Modifying the scoring algorithm for precision is not possible without re-validating the approach across retrospective datasets. An open area for additional research is a systematic approach for hardening, modifying, or adapting an existing model without negating the value of the prior clinical validation data.

## Supplementary Material

Table S1 Supplemental Table 1 includes the specific samples used for each experiment with the corresponding GES (RSI and 12CK GES) values.

Table S2 Supplemental Table 2 includes tissue/RNA assessment values.

## References

[1] van ’t Veer LJ , Dai H, van de Vijver MJ, He YD, Hart AAM, Mao M, . Gene expression profiling predicts clinical outcome of breast cancer. Nature 2002;415:530–6. 11823860 10.1038/415530a

[2] Erho N , Crisan A, Vergara IA, Mitra AP, Ghadessi M, Buerki C, . Discovery and validation of a prostate cancer genomic classifier that predicts early metastasis following radical prostatectomy. PLoS One 2013;8:e66855. 23826159 10.1371/journal.pone.0066855 PMC3691249

[3] Morris JS , Luthra R, Liu Y, Duose DY, Lee W, Reddy NG, . Development and validation of a gene signature classifier for consensus molecular subtyping of colorectal carcinoma in a CLIA-certified setting. Clin Cancer Res 2021;27:120–30. 33109741 10.1158/1078-0432.CCR-20-2403 PMC8713413

[4] Stewart JP , Richman S, Maughan T, Lawler M, Dunne PD, Salto-Tellez M. Standardising RNA profiling based biomarker application in cancer-The need for robust control of technical variables. Biochim Biophys Acta Rev Cancer 2017;1868:258–72. 28549623 10.1016/j.bbcan.2017.05.005

[5] Staunton JE , Slonim DK, Coller HA, Tamayo P, Angelo MJ, Park J, . Chemosensitivity prediction by transcriptional profiling. Proc Natl Acad Sci U S A 2001;98:10787–92. 11553813 10.1073/pnas.191368598 PMC58553

[6] Eschrich S , Zhang H, Zhao H, Boulware D, Lee J-H, Bloom G, . Systems biology modeling of the radiation sensitivity network: a biomarker discovery platform. Int J Radiat Oncol Biol Phys 2009;75:497–505. 19735874 10.1016/j.ijrobp.2009.05.056 PMC2762403

[7] Eschrich SA , Fulp WJ, Pawitan Y, Foekens JA, Smid M, Martens JWM, . Validation of a radiosensitivity molecular signature in breast cancer. Clin Cancer Res 2012;18:5134–43. 22832933 10.1158/1078-0432.CCR-12-0891 PMC3993974

[8] Strom T , Hoffe SE, Fulp W, Frakes J, Coppola D, Springett GM, . Radiosensitivity index predicts for survival with adjuvant radiation in resectable pancreatic cancer. Radiother Oncol 2015;117:159–64. 26235848 10.1016/j.radonc.2015.07.018 PMC7771365

[9] Ahmed KA , Chinnaiyan P, Fulp WJ, Eschrich S, Torres-Roca JF, Caudell JJ. The radiosensitivity index predicts for overall survival in glioblastoma. Oncotarget 2015;6:34414–22. 26451615 10.18632/oncotarget.5437 PMC4741462

[10] Torres-Roca JF , Fulp WJ, Caudell JJ, Servant N, Bollet MA, van de Vijver M, . Integration of a radiosensitivity molecular signature into the assessment of local recurrence risk in breast cancer. Int J Radiat Oncol Biol Phys 2015;93:631–8. 26461005 10.1016/j.ijrobp.2015.06.021 PMC5811194

[11] Strom T , Torres-Roca JF, Parekh A, Naghavi AO, Caudell JJ, Oliver DE, . Regional radiation therapy impacts outcome for node-positive cutaneous melanoma. J Natl Compr Cancer Netw 2017;15:473–82. 10.6004/jnccn.2017.0047 PMC7771284 28404758

[12] Sjöström M , Staaf J, Edén P, Wärnberg F, Bergh J, Malmström P, . Identification and validation of single-sample breast cancer radiosensitivity gene expression predictors. Breast Cancer Res 2018;20:64. 29973242 10.1186/s13058-018-0978-y PMC6033283

[13] Locati LD , Serafini MS, Iannò MF, Carenzo A, Orlandi E, Resteghin C, . Mining of self-organizing map gene-expression portraits reveals prognostic stratification of HPV-positive head and neck squamous cell carcinoma. Cancers (Basel) 2019;11:1057. 31357501 10.3390/cancers11081057 PMC6721309

[14] Mohammadi H , Prince A, Figura NB, Peacock JS, Fernandez DC, Montejo ME, . Using the radiosensitivity index (RSI) to predict pelvic failure in endometrial cancer treated with adjuvant radiation therapy. Int J Radiat Oncol Biol Phys 2020;106:496–502. 31759077 10.1016/j.ijrobp.2019.11.013 PMC7050205

[15] Kang B-H , Jang B-S, Kim IA. Radiosensitivity is associated with antitumor immunity in estrogen receptor-negative breast cancer. Breast Cancer Res Treat 2023;197:479–88. 36515748 10.1007/s10549-022-06818-7

[16] Torres-Roca JF , Erho N, Vergara IA, Davicioni E, Jenkins RB, Den R, . A molecular signature of radiosensitivity (RSI) is an RT-specific biomarker in prostate cancer. Int J Radiat Oncol Biol Phys 2014;90:S157.

[17] Scott JG , Berglund A, Schell MJ, Mihaylov I, Fulp WJ, Yue B, . A genome-based model for adjusting radiotherapy dose (GARD): a retrospective, cohort-based study. Lancet Oncol 2017;18:202–11. 27993569 10.1016/S1470-2045(16)30648-9 PMC7771305

[18] Ahmed KA , Liveringhouse CL, Mills MN, Figura NB, Grass GD, Washington IR, . Utilizing the genomically adjusted radiation dose (GARD) to personalize adjuvant radiotherapy in triple negative breast cancer management. EBioMedicine 2019;47:163–9. 31416721 10.1016/j.ebiom.2019.08.019 PMC6796536

[19] Scott JG , Sedor G, Ellsworth P, Scarborough JA, Ahmed KA, Oliver DE, . Pan-cancer prediction of radiotherapy benefit using genomic-adjusted radiation dose (GARD): a cohort-based pooled analysis. Lancet Oncol 2021;22:1221–9. 34363761 10.1016/S1470-2045(21)00347-8 PMC12818176

[20] Scott JG , Sedor G, Scarborough JA, Kattan MW, Peacock J, Grass GD, . Personalizing radiotherapy prescription dose using genomic markers of radiosensitivity and normal tissue toxicity in NSCLC. J Thorac Oncol 2021;16:428–38. 33301984 10.1016/j.jtho.2020.11.008 PMC8549863

[21] Coppola D , Nebozhyn M, Khalil F, Dai H, Yeatman T, Loboda A, . Unique ectopic lymph node-like structures present in human primary colorectal carcinoma are identified by immune gene array profiling. Am J Pathol 2011;179:37–45. 21703392 10.1016/j.ajpath.2011.03.007 PMC3123872

[22] Messina JL , Fenstermacher DA, Eschrich S, Qu X, Berglund AE, Lloyd MC, . 12-Chemokine gene signature identifies lymph node-like structures in melanoma: potential for patient selection for immunotherapy? Sci Rep 2012;2:765. 23097687 10.1038/srep00765 PMC3479449

[23] Raskin L , Fullen DR, Giordano TJ, Thomas DG, Frohm ML, Cha KB, . Transcriptome profiling identifies HMGA2 as a biomarker of melanoma progression and prognosis. J Invest Dermatol 2013;133:2585–92. 23633021 10.1038/jid.2013.197 PMC4267221

[24] Welsh EA , Eschrich SA, Berglund AE, Fenstermacher DA. Iterative rank-order normalization of gene expression microarray data. BMC Bioinformatics 2013;14:153. 23647742 10.1186/1471-2105-14-153 PMC3651355

[25] Berglund AE , Welsh EA, Eschrich SA. Characteristics and validation techniques for PCA-based gene-expression signatures. Int J Genomics 2017;2017:2354564. 28265563 10.1155/2017/2354564 PMC5317117

[26] Passing H. , Bablok. A new biometrical procedure for testing the equality of measurements from two different analytical methods. Application of linear regression procedures for method comparison studies in clinical chemistry, part I. J Clin Chem Clin Biochem 1983;21:709–20. 6655447 10.1515/cclm.1983.21.11.709

[27] Thiruthaneeswaran N , Bibby BAS, Pereira RR, More E, Denley H, Henry AM, . OC-1031: the radiosensitivity index predicts benefit from HDR brachytherapy in high-risk prostate cancer. Radiother Oncol 2020;152:S1086–7.

[28] Ahmed KA , Fulp WJ, Berglund AE, Hoffe SE, Dilling TJ, Eschrich SA, . Differences between colon cancer primaries and metastases using a molecular assay for tumor radiation sensitivity suggest implications for potential oligometastatic SBRT patient selection. Int J Radiat Oncol Biol Phys 2015;92:837–42. 25838188 10.1016/j.ijrobp.2015.01.036 PMC4481172

[29] Yang G , Yuan Z, Ahmed K, Welsh EA, Fulp WJ, Gonzalez RJ, . Genomic identification of sarcoma radiosensitivity and the clinical implications for radiation dose personalization. Transl Oncol 2021;14:101165. 34246048 10.1016/j.tranon.2021.101165 PMC8274330

[30] Johnstone PAS , Spiess PE, Sedor G, Grass GD, Yamoah K, Scott JG, . Changing radiotherapy paradigms in penile cancer. Eur Urol Open Sci 2022;36:47–8. 35028598 10.1016/j.euros.2021.12.005 PMC8739469

[31] Strom T , Harrison LB, Giuliano AR, Schell MJ, Eschrich SA, Berglund A, . Tumour radiosensitivity is associated with immune activation in solid tumours. Eur J Cancer 2017;84:304–14. 28863385 10.1016/j.ejca.2017.08.001 PMC5822441

[32] Khan MT , Yang L, More E, Irlam-Jones JJ, Valentine HR, Hoskin P, . Developing tumor radiosensitivity signatures using LncRNAs. Radiat Res 2021;195:324–33. 33577642 10.1667/RADE-20-00157.1

[33] Ahmed KA , Scott JG, Arrington JA, Naghavi AO, Grass GD, Perez BA, . Radiosensitivity of lung metastases by primary histology and implications for stereotactic body radiation therapy using the genomically adjusted radiation dose. J Thorac Oncol 2018;13:1121–7. 29733909 10.1016/j.jtho.2018.04.027 PMC7810135

[34] Ahmed KA , Berglund AE, Welsh EA, Naghavi AO, Kim Y, Yu M, . The radiosensitivity of brain metastases based upon primary histology utilizing a multigene index of tumor radiosensitivity. Neuro Oncol 2017;19:1145–6. 28379582 10.1093/neuonc/nox043 PMC5570253

[35] Prabhakaran S , Rizk VT, Ma Z, Cheng C-H, Berglund AE, Coppola D, . Evaluation of invasive breast cancer samples using a 12-chemokine gene expression score: correlation with clinical outcomes. Breast Cancer Res 2017;19:71. 28629479 10.1186/s13058-017-0864-z PMC5477261

[36] Li R , Berglund A, Zemp L, Dhillon J, Putney R, Kim Y, . The 12-CK score: global measurement of tertiary lymphoid structures. Front Immunol 2021;12:694079. 34267760 10.3389/fimmu.2021.694079 PMC8276102

[37] Li X , Wan Z, Liu X, Ou K, Yang L. A 12-chemokine gene signature is associated with the enhanced immunogram scores and is relevant for precision immunotherapy. Med Oncol 2022;39:43. 35092511 10.1007/s12032-021-01635-2

[38] Xu W , Ma C, Liu W, Anwaier A, Tian X, Shi G, . Prognostic value, DNA variation and immunologic features of a tertiary lymphoid structure-related chemokine signature in clear cell renal cell carcinoma. Cancer Immunol Immunother 2022;71:1923–35. 35043231 10.1007/s00262-021-03123-y PMC10992571

